# Genome-Wide Identification and Expression Analysis of the *PUB* Gene Family in *Zoysia japonica* under Salt Stress

**DOI:** 10.3390/plants13060788

**Published:** 2024-03-10

**Authors:** Daojin Sun, Jingya Xu, Haoran Wang, Hailin Guo, Yu Chen, Ling Zhang, Jianjian Li, Dongli Hao, Xiang Yao, Xiaohui Li

**Affiliations:** 1 The National Forestry and Grassland Administration Engineering Research Center for Germplasm Innovation and Utilization of Warm-Season Turfgrasses, Institute of Botany, Jiangsu Province and Chinese Academy of Sciences (Nanjing Botanical Garden Mem. Sun Yat-Sen), Nanjing 210014, China; sundaojin@jib.ac.cn (D.S.); guohailin@jib.ac.cn (H.G.);; 2 Jiangsu Key Laboratory for the Research and Utilization of Plant Resources, Institute of Botany, Jiangsu Province and Chinese Academy of Sciences (Nanjing Botanical Garden Mem. Sun Yat-Sen), Nanjing 210014, China; 3 College of Agro-Grassland Science, Nanjing Agricultural University, Nanjing 210095, China

**Keywords:** *PUB* gene family, genome-wide analysis, qRT-PCR, salt stress, transcriptome data, *Zoysia japonica*

## Abstract

The U-box protein family of ubiquitin ligases is important in the biological processes of plant growth, development, and biotic and abiotic stress responses. Plants in the genus *Zoysia* are recognized as excellent warm-season turfgrass species with drought, wear and salt tolerance. In this study, we conducted the genome-wide identification of *plant U-box* (*PUB*) genes in *Zoysia japonica* based on U-box domain searching. In total, 71 *ZjPUB* genes were identified, and a protein tree was constructed of AtPUBs, OsPUBs, and ZjPUBs, clustered into five groups. The gene structures, characteristics, *cis*-elements and protein interaction prediction network were analyzed. There were mainly ABRE, ERE, MYB and MYC *cis*-elements distributed in the promoter regions of *ZjPUBs*. *ZjPUBs* were predicted to interact with PDR1 and EXO70B1, related to the abscisic acid signaling pathway. To better understand the roles of *ZjPUBs* under salt stress, the expression levels of 18 *ZjPUBs* under salt stress were detected using transcriptome data and qRT-PCR analysis, revealing that 16 *ZjPUBs* were upregulated in the roots under salt treatment. This indicates that *ZjPUBs* might participate in the *Z. japonica* salt stress response. This research provides insight into the *Z. japonica PUB* gene family and may support the genetic improvement in the molecular breeding of salt-tolerant zoysiagrass varieties.

## 1. Introduction

The ubiquitin-proteasome system (UPS) is one of the most important pathways for the selective degradation of proteins in eukaryotic cells, and it plays an important role in the regulation of cellular functions [1]. The UPS consists of the ubiquitin protein (Ub), ubiquitin-activating enzyme (E1), ubiquitin-conjugating enzyme (E2), ubiquitin ligase (E3), and the complete 26S proteasome. During the ubiquitination process, E1 first activates ubiquitin, which is then transferred to E2, and finally ubiquitin is added to specific target proteins via E3 ligase. Target proteins bound to polyubiquitin chains are usually degraded by the 26S proteasome, whereas monoubiquitinated proteins tend to regulate protein activity [2,3]. The E3 ubiquitin ligases provide substrate recognition and binding specificity, which determine protein ubiquitination specificity. In addition, E3s can regulate the expression levels of transcription factors associated with plant responses to adversity stresses such as salinity, drought and freezing damage [4,5,6].

Based on their mechanism of action and specific structural domains, E3 ligases can be classified into two main classes: single-subunit and multi-subunit types. The Homologous to E6-associated protein Carboxyl Terminus (HECT) and Really Interesting New Gene (RING) finger and U-box domains are categorized into the single-subunit type, while Skp1-Cullin-F-box (SCF), Anaphase-Promoting Complex (APC), and VHL-Elongin B-Elongin C (VBC) are classified as the multi-subunit type [7,8,9]. Currently, plant U-box type E3 ubiquitin ligases, as members of the single-subunit group, are widely distributed in eukaryotic organisms. The U-box domain contains about 70 amino acids, which is structurally related to RING-type [10]. Plant U-box (PUB) proteins are identified in an increasing number of plant species with different gene numbers. Compared to the two and 21 U-box genes found in yeast and human genomes [11], the number of *PUB* genes in plants is much higher. For example, there are 77 *PUB* genes in rice [12], 64 in Arabidopsis [13], 59 in *Sorghum bicolor* [14] and 62 in Chinese white pear [15]. *PUB* genes in plants have been reported to have distinct functions in plant growth, development, and resistance to biotic and abiotic stress [16,17]. For the intracellular processes, the kinase-PUB pattern has been reported to regulate intracellular protein hydrolysis. Moreover, PUB protein can regulate pollen self-incompatibility by associating with cell membrane components [16]. In addition, *PUB* genes play important roles in the response to high salt. Studies have shown that *AtPUB18* and *AtPUB19* are induced by salt stress in Arabidopsis and the *pub18pub19* double mutant exhibits salt sensitivity compared to the wild type during the seed germination stage [18]. In strawberry (*Fragaria × ananassa* Duch.), the *PUB* genes *FaU-box83*, *FaU-box3*, *FaU-box98* and *FaU-box136* are continuously induced by salt stress [10]. Overexpression of wheat (*Triticum aestivum* L.) *TaPUB26* in *Brachypodium distachyon* disrupts intracellular ion homeostasis while decreasing the activity of antioxidant enzymes, which in turn reduces the salt tolerance of transgenic *B. distachyon* [19]. Overexpression of the *TaPUB1* gene in wheat can maintain a low Na^+^/K^+^ ratio and regulate antioxidant enzyme activity to improve salt tolerance in transgenic wheat [19]. Although the biological functions of *PUB* genes with regard to salt stress tolerance have been reported in some plants [10,14,20], the functions of *PUB* genes have not yet been reported in perennial halophytes, and the regulation mechanism of *PUB* genes in response to salt stress in different plants remains to be further elucidated.

*PUB* genes are widely distributed in plants and have now been identified in Arabidopsis [12], rice [13], sorghum [14], and Chinese white pear [15]. However, these genes have not previously been reported in plants of the genus *Zoysia*. Salt stress is a major abiotic stress in plants, resulting in enormous losses in grain yield [21]. The genus *Zoysia* is recognized as a perennial and excellent warm-season turfgrass with good drought and salt tolerance worldwide. Numerous studies have assessed the effects of salinity and revealed that zoysiagrass is the most salt tolerant of the C4 grass species in the family Poaceae. *Zoysia japonica*, *Zoysia matrella*, and *Zoysia macrostachya* are classified as halophytes [22,23,24], displaying stronger salt tolerance than most plants. Halophytes are reported to have evolved specialized strategies to cope with high salinity stress [25,26]. Understanding the salt tolerance mechanism in halophytes may provide new insights into plant salt tolerance. Studies on salt tolerance in the halophyte *Z. japonica* have generated a great deal of interest since the 1990s. Currently, the studies on the molecular mechanisms of salt tolerance in *Zoysia* plants are mainly associated with the identification of genes and their biological function [27,28,29,30]. To date, the U-box E3 ubiquitin ligase genes have not been studied in *Z. japonica*, although *PUB* genes have been reported to be associated with salt tolerance in many other plants [10,17]. The present study conducted the genome-wide identification, *cis*-element analysis, and protein interaction prediction of *PUB* genes under salt stress in *Z. japonica*, and investigated their evolutionary relationships, gene structures, and expression patterns. The findings of this study could help elucidate the putative roles of *PUB* genes in the salt tolerance of *Z. japonica* and predict the corresponding regulatory networks of *ZjPUBs*.

## 2. Results

### 2.1. Identification and Protein Tree Construction of the PUB Gene Family in Z. japonica

**Figure 1:**
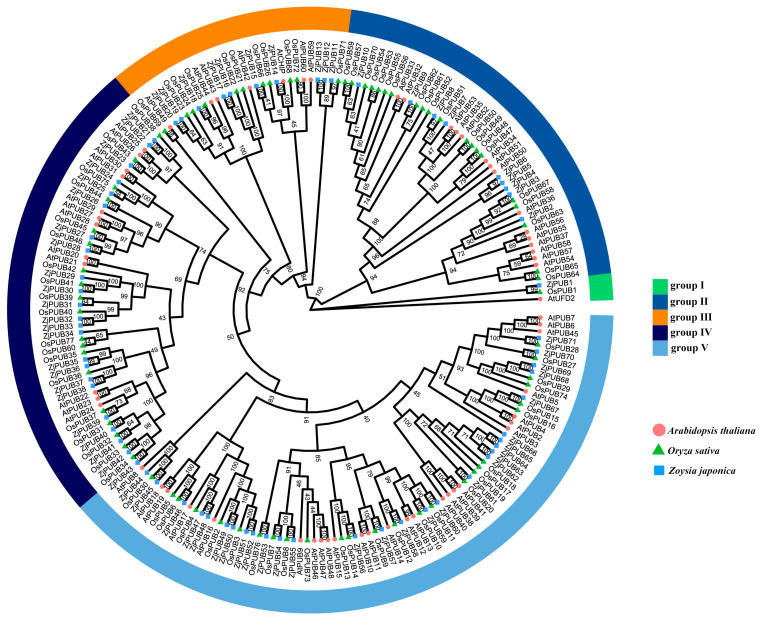
Protein tree construction of AtPUBs, OsPUBs, and ZjPUBs using the maximum likelihood method with 1000 bootstrap values. Different shapes on branches represent corresponding species, of which the circle shape in pink, triangle shape in green and rectangle shape in blue represent *Arabidopsis thaliana*, *Oryza sativa* and *Zoysia japonica*, respectively. Different colors outside the protein tree indicate corresponding PUB groups, of which the green color, blue color, orange color, midnight blue color and sky-blue color represent group I, II, III, IV and V, respectively.

A total of 71 *PUB* genes were identified in *Z. japonica* Steud. This study constructed a protein tree with AtPUB in Arabidopsis, OsPUB in rice and ZjPUB protein sequences (Figure 1). In previous studies, *OsPUB2*, *OsPUB3* [31] and *OsPUB67* [32] in *Oryza sativa* and *AtPUB18*, *AtPUB19* [18] and *AtPUB30* [33] in *Arabidopsis thaliana* were reported to be related to salt stress. In this study, *ZjPUB* genes were designated as *ZjPUB1* through *ZjPUB71* according to their order in the protein tree, due to the lack of a chromosome-level genome. As shown in Figure 1, the topology of the protein tree was divided into five groups. Group I was the smallest with three proteins (AtUFD2, OsPUB1 and ZjPUB1), indicating closer evolutionary relationships among these three proteins. There were 45 proteins in group II, of which ZjPUB3 showed a closer evolutionary relationship with OsPUB67. There were 28 proteins in group III, of which ZjPUB14 was closely clustered with AtCHIP. There were 53 proteins in group IV, of which AtPUB30, OsPUB75, ZjPUB24 and ZjPUB25 were closely clustered. Group V was the largest with 81 proteins, of which AtPUB18, AtPUB19, OsPUB5, OsPUB6 and ZjPUB46 were closely clustered. According to the constructed protein tree, OsPUB2, OsPUB3, ZjPUB49, ZjPUB50, ZjPUB51, and ZjPUB52 appeared to be closely related according to the protein tree. In different groups on the protein tree, the PUB proteins of *Z. japonica* and rice showed a closer relationship with those of rice, consistent with their evolutionary relationship.

### 2.2. Characteristics, Gene Structure and Domain Analysis of PUB Genes in Z. japonica

The basic characteristics of 71 *ZjPUBs* were predicted and analyzed, including transcript ID, accession number of AtPUB orthologues, coding sequence (CDS) length, the amino acids (aa) length of ZjPUB protein sequences, theoretical isoelectric point (pI), protein molecular weight (MW), and subcellular localization, as shown in Table 1. The shortest ZjPUB was ZjPUB15 with 101 aa, while the longest ZjPUB was ZjPUB1 with 2,967 aa. The average length of all ZjPUBs was 644 aa. This indicates that the length of protein sequences of *ZjPUBs* is different. The MW values ranged from 11.51 kDa (ZjPUB15) to 112.71 kDa (ZjPUB16), with an average of 60.87 kDa. The pI values ranged from 4.55 (ZjPUB12) to 10.25 (ZjPUB31). Subcellular localization analysis showed that 53 ZjPUBs were predicted to localize in the nucleus, 11 ZjPUBs were predicted to localize in the chloroplast, three ZjPUBs were predicted to localize in the endomembrane system, and only one ZjPUB each was predicted to localize in chloroplast outer membrane, chloroplast thylakoid membrane, mitochondrion, and plasma membrane.

**Figure 2:**
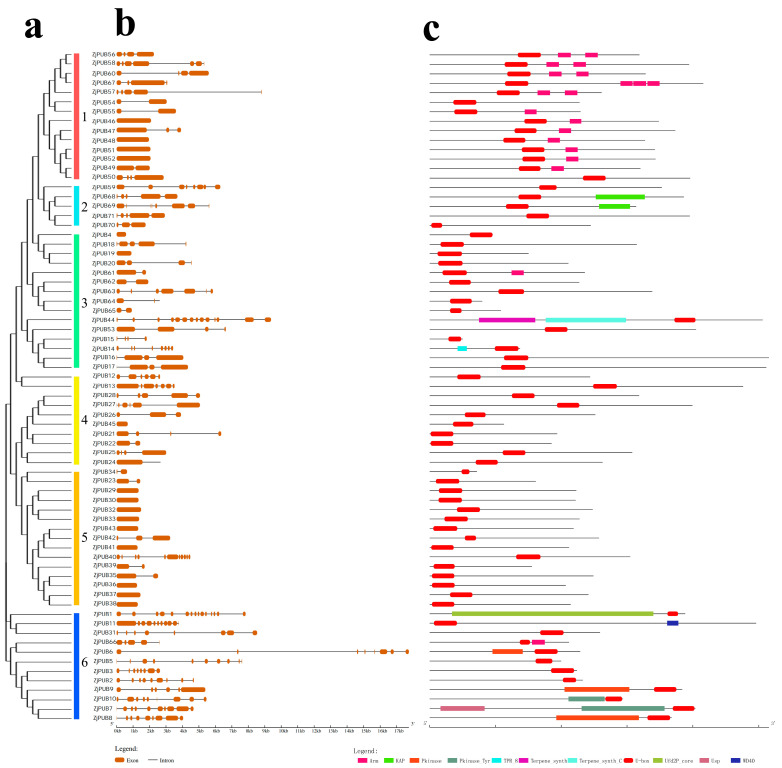
Schematic representation of the protein tree, gene structure and domain analysis of 71 *ZjPUBs*. (**a**) Protein tree construction of 71 ZjPUBs using the maximum likelihood method with the protein sequences of ZjPUBs, and different colors indicate different PUB groups. (**b**) Gene structure representation of *ZjPUBs*. The rectangles represent exon and lines represent the introns. (**c**) Protein domain representation of 71 ZjPUBs. Different colors represent different conserved domains.

Gene structure and protein domain analysis of ZjPUBs were performed to better understand the composition and function of the *ZjPUB* genes. As shown in Figure 2a, the protein sequences of 71 ZjPUBs were used to construct a protein tree with the maximum likelihood method, divided into six groups. The exon number of *ZjPUBs* varied from 1 to 15, indicating that there might be complex RNA splicing processes in *ZjPUB* genes (Figure 2b). ZjPUBs in group 5 had similar evolutionary relationships, and most had only one exon, while ZjPUBs in group 6 had more exons. Except for a U-box protein domain, ZjPUB proteins also had other domains, such as ARM, KAP, Pkinase, and WD40 (Figure 2c). In group 1, except for ZjPUB50 and ZjPUB54, all 13 ZjPUBs had ARM protein domain. ZjPUB56, 57, 58, 60 and 67 had ARM repeat domain, and ZjPUB46, 47, 48, 49, 50, 51, 52 and 55 had only one ARM domain. In group 2, ZjPUB68 and ZjPUB69 contained the KAP protein domain. In group 3, there were both Terpene_syhth and Terpene_syhth_C protein domains in ZjPUB44. Although there were different exon numbers in both group 4 and 5, all ZjPUBs of these two groups only possessed the U-box domain, confirming the close evolutionary relationship between group 4 and group 5. In group 6, only ZjPUB33 had WD40 protein domain.

### 2.3. Cis-Acting Regulatory Element Prediction in Promoter Regions of ZjPUB Family Members

**Figure 3:**
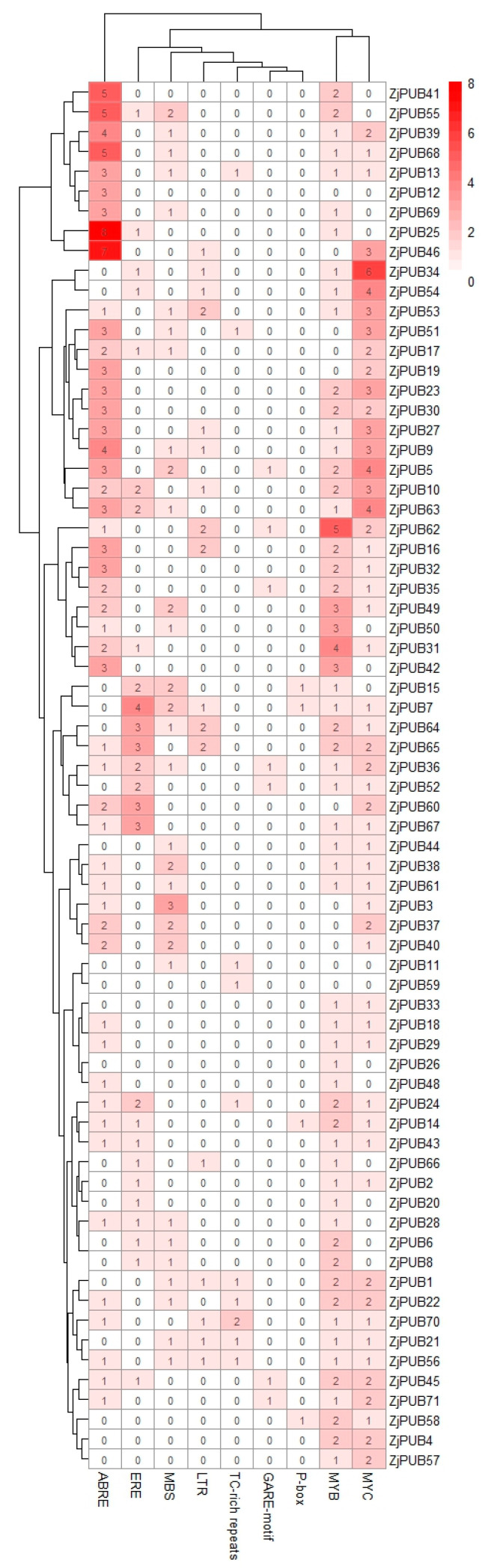
*Cis*-element prediction of *PUB* gene family members of *Zoysia japonica*. The colors and numbers in the box represent the corresponding frequency of *cis*-elements in the promoter regions of *ZjPUBs*.

To better understand the regulatory relationships between *ZjPUBs* and other transcription factors, the promoter regions of 71 *PUB* genes of *Z. japonica* were analyzed and predicted with conserved *cis*-elements (Figure 3). Nine stress-related *cis*-elements were selected, including abscisic acid responsive element (ABRE), ethylene responsive element (ERE), gibberellin-responsive element (GARE-motif, P-box), anaerobic induction element (ARE), low temperature responsive element (LTR), defense and stress responsive element (TC-rich), MYB-related element (MBS and MYB), and MYC. Obviously, ABRE motif was most abundant in the promoter regions of *ZjPUBs*, indicating abscisic acid (ABA) might be widely involved in the transcription regulation of *ZjPUBs*. Among them, the *ZjPUB25* promoter region had the maximum number of eight ABRE motifs, followed by *ZjPUB46* containing seven ABRE motifs. Furthermore, MBS, MYB and MYC *cis*-elements were also distributed in the promoter regions of *ZjPUBs*, indicating the regulatory roles of MYB and MYC. Eight *ZjPUBs* (*ZjPUB15*, *ZjPUB7*, *ZjPUB64*, *ZjPUB65*, *ZjPUB36*, *ZjPUB52*, *ZjPUB60* and *ZjPUB67*) had more ERE cis-elements in the promoter regions. This indicated that ethylene might play regulatory roles in the transcription regulation of *ZjPUBs*. Fewer GARE-motif and P-box elements were found in the promoter regions of *ZjPUBs*, suggesting that gibberellic acid (GA) might possess an unimportant role in the transcription regulation of *ZjPUBs*. The transcription regulation of *ZjPUBs* might be mainly related to ABA, ethylene, MYB and MYC transcription factors, indicating corresponding regulatory relationships.

### 2.4. Expression Analysis of ZjPUB Genes under Salt Stress

**Figure 4:**
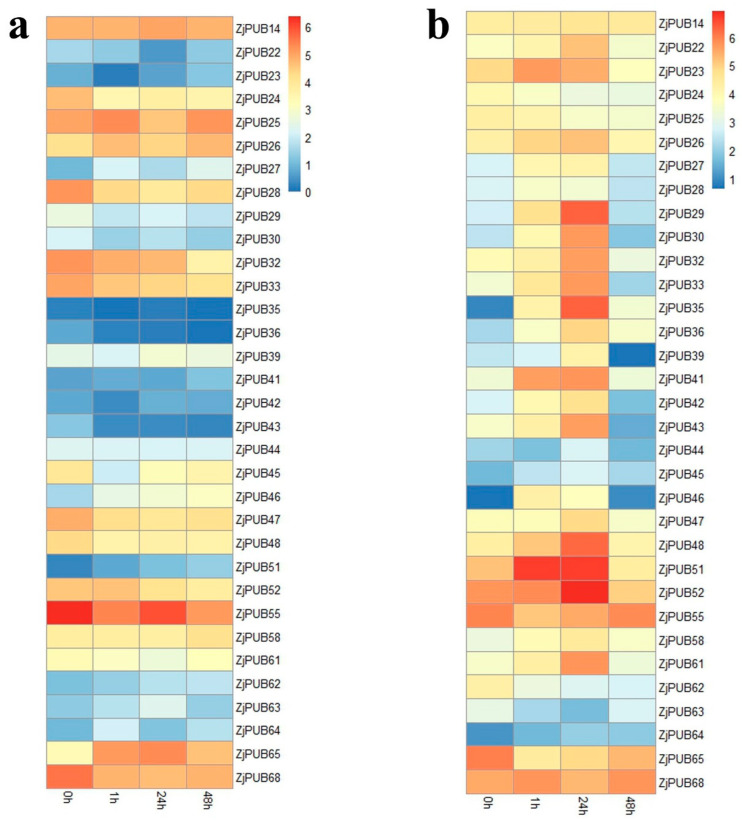
Heatmap of the expression profiles of *PUB* genes of *Zoysia japonica* at different time points after salt stress. Expression levels were normalized by log2. (**a**) Expression profiles in the leaves, at 0 h, 1 h, 24 h and 72 h after salt stress. (**b**) Expression profiles in the roots, at 0 h, 1 h, 24 h and 72 h after salt stress.

Based on previous transcriptome data of *Z. japonica* ‘Z011′ from 0 h to 48 h under salt stress [34], the expression levels of 33 *ZjPUB* genes with fragments per kilobase of transcript per million mapped reads (FPKM) > 1 were analyzed in roots and leaves (Figure 4). After salt treatment, the expression levels of most *ZjPUBs* in leaves showed no significant changes except for *ZjPUB46*, *51*, *63*, *64*, and *65*, which were upregulated after salt treatment. Among them, *ZjPUB64* was upregulated at 1 h after the beginning of salt stress (3.27-fold change), and downregulated at 24 h and 72 h compared to 1h. This finding indicated that *ZjPUB64* might participate in short-term salt stress response in the leaves of *Z. japonica*. The expression levels of *ZjPUB46* and *ZjPUB51* were upregulated continuously from 0 h to 24 h after salt stress (3.91-fold change and 8.39-fold change, respectively). The expression levels of *ZjPUB63* and *ZjPUB65* reached the maximum at 24 h after salt treatment (2.74-fold change and 4.31-fold change, respectively).

In the roots, 20 *ZjPUB* genes (*ZjPUB22*, *ZjPUB26*, *ZjPUB27*, *ZjPUB29*, *ZjPUB30*, *ZjPUB32*, *ZjPUB33*, *ZjPUB35*, *ZjPUB36*, *ZjPUB39*, *ZjPUB41*, *ZjPUB42*, *ZjPUB43*, *ZjPUB46*, *ZjPUB47*, *ZjPUB48*, *ZjPUB52*, *ZjPUB58*, *ZjPUB61* and *ZjPUB64*, from 2- to 85-fold change) were obviously upregulated after salt treatment at 24 h in total, compared to 0 h. The expression patterns in the two organs indicated that *ZjPUB* genes might play major roles in salt-response processes of roots.

**Figure 5:**
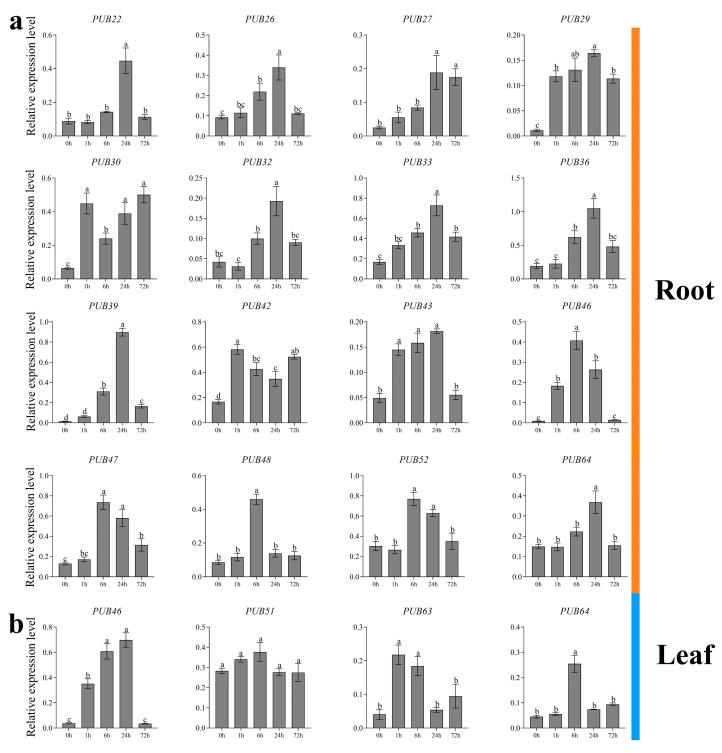
Relative expression levels of *ZjPUB* genes after salt stress in roots (**a**) and leaves (**b**). Error bars indicate the standard deviation (SD) for three biological replicates based on qRT-PCR, and shared letters indicate no statistically significant difference between the means (*p* > 0.05) as determined by ANOVA.

qRT–PCR was performed to verify the FPKM values of *ZjPUB* genes in roots and leaves under salt stress at 0 h, 1 h, 6 h, 24 h and 48 h (Figure 5). All 16 *ZjPUBs* in roots showed upregulated expression levels after salt treatment, indicating that *ZjPUBs* might participate in salt-response regulation. The expression levels of *ZjPUB22*, *ZjPUB26*, *ZjPUB27*, *ZjPUB29*, *ZjPUB32*, *ZjPUB33*, *ZjPUB36*, *ZjPUB39*, *ZjPUB43* and *ZjPUB64* gradually increased over time after salt treatment and reached the maximum values at 24 h (Figure 5a). The expression levels of *ZjPUB46*, *ZjPUB47*, *ZjPUB48* and *ZjPUB52* reached the maximum values at 6 h after salt stress.

Expression levels of four *ZjPUB* genes (*ZjPUB46*, *ZjPUB51*, *ZjPUB63* and *ZjPUB64*) in leaves were also measured (Figure 5b). There were no significant changes in the expression of *ZjPUB51*. The expression levels of *ZjPUB46*, *ZjPUB63* and *ZjPUB64* reached the peaks at 24 h, 1 h and 6 h after the onset of salt stress, respectively. Most of the expression patterns of *ZjPUBs* in the relative expression analysis were in line with those in transcriptome data. Based on transcriptome data and qRT-PCR, this study found that *ZjPUBs* were mainly upregulated in the roots, indicating their potential regulatory function in salt tolerance.

### 2.5. Protein–Protein Interaction (PPI) Network Analysis of Differentially Expressed ZjPUB Members under Salt Stress

**Figure 6:**
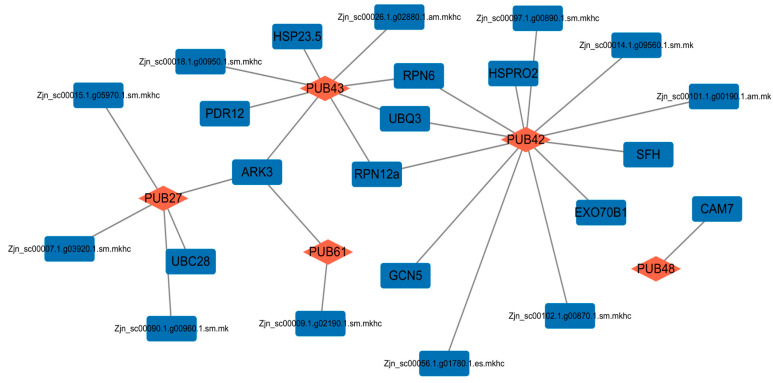
PPI network of ZjPUB27, ZjPUB42, ZjPUB43, ZjPUB48 and ZjPUB61. Orange diamonds represent ZjPUB proteins and blue rectangles represent other predicted proteins.

To predict the function pattern of ZjPUBs, the PPI network was constructed based on the orthologues in *A. thaliana*. Eighteen ZjPUBs were predicted to interact with other proteins in total, which are shown in Table S1. Five differentially expressed ZjPUBs were used to construct the PPI network shown in Figure 6. ZjPUB43 was predicted to interact with HSP23.5, PDR12, ARK3, RPN12a, UBQ3, and RPN6 in the network. ZjPUB42 was predicted to interact with HSPRO2, RPN6, UBQ3, RPN12a, EXO70B1, and SFH. ZjPUB27 was predicted to interact with UBC28, and ZjPUB61 was predicted to interact with ARK3 as well. ZjPUB48 was predicted to interact with CAM7, which encodes a calmodulin.

## 3. Discussion

Protein ubiquitination occupies profound roles in cellular pathways across eukaryotes, regulating biological processes through posttranslational modification, in which E3 ubiquitin ligases are crucial [35]. *PUB* genes have a conserved U-box motif consising of about 70 aa, which regulates the ubiquitination of substrates [8]. In this study, 71 *ZjPUB* genes were identified and analyzed with evolutionary relationships, gene structures and protein domains. Based on the protein tree, evolutionary relationships of PUBs between *Z. japonica* and rice were closer, and the *PUB* gene numbers of those were also similar, which were 71 and 77, respectively [36]. In the protein tree, AtUFD2, OsPUB1 and ZjPUB1 were clustered into group I (Figure 1). Similar to AtUFD2, ZjPUB1 also had both U-box domain at C-terminus and UFD2 core domain at N-terminus (Figure 2). AtUFD2 contains a conserved domain similar to that of UFD2 in yeast and can intearact with CDC48 protein, regulating the cell cycle, death, and other physiological activities [1]. ZjPUB1 might also regulate these processes, due to the similar protein structure. In addition to the UFD2 core domain, there were ARM, KAP, Pkinase, TPR, USP, Terpene and WD40 found in ZjPUBs. In group V of the protein tree, AtPUB18, AtPUB19, OsPUB5, OsPUB6 and ZjPUB46 were closely clustered, and OsPUB3 showed a closer relationship with ZjPUB52 (Figure 1). *OsPUB2* was upregulated by high salinity, drought, and cold [31], and *pub18pub19* double mutants displayed reduced salt sensitivity in Arabidopsis [18]. *ZjPUB46* and *ZjPUB52* were upregulated significantly at 6 h in roots after salt stress (Figure 5a), indicating similar biological functions.

As shown in Figure 2, there were ARM motifs at the C-terminus in 14 ZjPUBs, of which five ZjPUBs (ZjPUB56, ZjPUB 57, ZjPUB58, ZjPUB60 and ZjPUB67) in group 1 had ARM repeat domains. ARM repeats primarily mediate the interaction between PUB proteins and their substrates, making the substrates available for ubiquitination [21,37]. Of the PUB proteins with ARM repeats in Arabidopsis, *pub18pub19* double mutants were reported to be related to salt stress [18], and *AtPUB16* and *AtPUB17* were reported to play a role in plant defense [38,39]. The 14 ZjPUBs with U-box and ARM domains were clustered in group V with AtPUB16, AtPUB17, AtPUB18 and AtPUB19, indicating similar biological functions. In group 6, there were TRR, USP and Pkinase domains in ZjPUBs, which might be related to signal transduction via phosphorylation in the cellular processes [14].

By performing the analysis on PLANTCARE [40], ABRE, ERE, MYB and MYC motifs were found to be distributed in the promoter regions of *ZjPUB* genes, indicating that *ZjPUBs* might be induced by ABA, ethylene, MYB and MYC transcription factors. In Arabidopsis, the expression levels of *AtPUB19* were induced by ABA, and the overexpression of *AtPUB19* resulted in reduced plant sensitivity to ABA and hypersensitivity to dehydration [41]. In soybean (*Glycine max*), the expression of *GmPUB8* was upregulated by exogenous ABA and NaCl, and the overexpression of *GmPUB8* in Arabidopsis showed decreased drought tolerance and enhanced sensitivity of osmotic and salt stress [42]. MdPUB24, an Ethylene-activated PUB protein in apple (*Malus domestica*), directly interacted with and ubiquitinated MdBEL7 which repressed the expression of chlorophyll catabolic genes, resulting in the degreening of apple fruits [43]. The MYB transcription factors have been reported to participate in the responses to abiotic and biotic stress, such as the drought response [44], salt stress response [45], and cold response [46]. The MYB-binding site is involved in flavonoid biosynthesis, related to stress responses in plants [47]. MYC transcription factors are involved in jasmonic acid (JA) signaling pathway, regulating the plant tolerance to abiotic stress such as oxidative stress [48]. Therefore, the predicted motifs in the promoters of *ZjPUBs* might be involved in a complex network together with ABA, ethylene and JA hormones to regulate both abiotic and biotic stress responses.

In the PPI network, ZjPUB proteins were predicted to interact with EXO70B1 and PDR12, which could participate in the ABA signaling pathway. EXO70B1 is reported to be a subunit of exocyst, which regulates stomatal closure induced by ABA [49]. In grapevine (*Vitis vinifera*), VviPUB19 ubiquitinated and degraded VviExo70B and the overexpression of VviPUB19 in grape callus and Arabidopsis reduced the drought and NaCl tolerance and increased the sensitivity to ABA, which was opposite to the phenotype of VviExo70B overexpression plants [50]. PDR12 (ABCG40) is a plasma membrane ABA uptake transporter [51], which is predicted to interact with ZjPUB42. *PUB* genes were found to play important roles in the ABA signaling pathway [52]. PUB12/13 E3 ligases ubiquitinated and degraded the ABA co-receptor ABI1, affecting ABA responses in Arabidopsis [53]. Therefore, *ZjPUB* genes in *Z. japonica* might be activated by ABA, and may also regulate ABA signaling pathways via ubiquitination.

Studies have shown that *PUB* genes are widely involved in salt stress responses in plants. For example, *TaPUB1*-overexpressed wheat plants had a lower Na^+^/K^+^ ratio and enhanced antioxidant enzyme activities under salt stress [11]; and *Ospub15* mutants in rice caused growth retardation and lethal phenotypes in seedlings, while *OsPUB15*-overexpressed rice plants had higher salt tolerance than the wild type [54]. In wheat, *TaPUB15* was expressed in various tissues, but the expression level of *TaPUB15* in roots was significantly higher compared to other tissues, and the overexpression of *TaPUB15* improved salt tolerance in transgenic rice plants [55]. *Z. japonica* is one of the most salt-tolerant halophytes, whose molecular mechanisms of salt tolerance are of great importance. The identification of the response of *ZjPUB* genes to salt stress contributes to the further study of the molecular mechanism underlying salt tolerance in *Z. japonica*. Furthermore, because the root is the primary organ for plants to perceive soil stress signals, root characteristics might determine the stress resistance of plants [15]. In this study, the expression levels of 16 *ZjPUBs* in roots increased significantly compared to those in leaves, indicating that *ZjPUBs* play crucial roles in the root response to salt stress in *Z. japonica*. This has important implications for the genetic improvement of highly salt-tolerant zoysiagrass species, meeting economic needs and improving saline soil environment.

## 4. Materials and Methods

### 4.1. Genome and Transcriptome Data Sources

The reference genome data of *Z. japonica* was from the Zoysia Genome Database (http://zoysia.kazusa.or.jp, accessed on 7 November 2023) [56]. The transcriptome data of *Z. japonica* Z011 was from sequenced data in our previous study (NCBI accession number: PRJNA559944, https://www.ncbi.nlm.nih.gov/bioproject/PRJNA559944/, accessed on 7 November 2023), and analyzed following the same procedure as that described by Wang et al. [34].

### 4.2. Identification of ZjPUBs and Construction of the Protein Tree

The protein database of *Z. japonica* was obtained from the Zoysia Genome Database (http://zoysia.kazusa.or.jp, accessed on 7 November 2023) [56]. The seed file of U-box domain (PF04564) was used to search the candidate *PUB* genes in the *Z. japonica* protein database using Pfam_scan software (E-value ≤ 10^−5^) (version 14.0, https://github.com/SMRUCC/GCModeller/tree/master/src/interops/scripts/PfamScan, accessed on 20 October 2023) [57]. ZjPUB protein sequences are shown in Table S2.

The protein sequences of AtPUBs were retrieved from The Arabidopsis Information Resource (TAIR) database (https://www.arabidopsis.org/browse/genefamily/plantubox.jsp, accessed on 22 Octorber 2023) and OsPUBs were obtained from the Rice Genome Annotation Project (http://rice.uga.edu/index.shtml, accessed on 22 October 2023) [58]. In total, 209 protein sequences of AtPUBs, OsPUBs and ZjPUBs were aligned with the Muscle function using MEGAX software (version 10.1.7, https://www.megasoftware.net/, accessed on 22 October 2023) [59]. The protein tree was constructed using iqtree [60] (maximum likelihood method with the VT+F+R7 model) and the bootstrap values were set to 1000. The evolutionary tree was visualized using the R package ‘ggtree’ v3.8.2 [61].

### 4.3. Characteristics, Gene Structure and Domain Analysis of PUB Genes in Z. japonica

The MW values, aa number, and PI values of 71 ZjPUBs were predicted using ExPASy [62]. The protein domains were identified using the Pfam database (http://pfam.xfam.org/, accessed on 20 October 2023) [57] and the gene structure (introns-exons) was analyzed using the genome annotation files in the Zoysia Genome Database [56], which was visualized by GSDS2.0 (http://gsds.gao-lab.org/, accessed on 10 December 2023) [63]. Subcellular localization was predicted using BUSCA (https://busca.biocomp.unibo.it/, accessed on 11 December 2023) [64].

### 4.4. Cis-Acting Elements within the Promoter Region of ZjPUB Genes

The 2000 bp upstrem of the coding sequence (CDS) was considered to be the promoter region of each *ZjPUB* gene. These promoter regions were extracted with TBtools (version 2.034, https://github.com/CJ-Chen/TBtools-II, accessed on 10 December 2023) [65]. The extracted promoter sequences were submitted to the online website PlantCARE (http://bioinformatics.psb.ugent.be/webtools/plantcare/html/, accessed on 12 December 2023) for *cis*-acting element prediction [40]. The heatmap of *cis*-element distribution in promoter regions of *ZjPUBs* was visualized using the R package ‘pheatmap’ v1.0.12 [66].

### 4.5. Gene Expression Analysis of ZjPUBs under Salt Stress

Expression levels of *PUB* genes of *Z. japonica* Steud. Z011 under 350 mM NaCl treatment at 0 h, 1 h, 24 h, and 72 h were retrieved from the transcriptome analysis in our previously published work [34]. Expression of *ZjPUBs* was calculated with FPKM values. Compared to 0 h, fold changes of FPKM values of each time points were normalized with log2 (fold change + 1), which was visualized by the R package ‘pheatmap’ v1.0.12 [66].

For the qRT-PCR analysis, stolons of *Z. japonica* Steud. Z011 were obtained and cultivated using hydroponics according to the methods established in our previous study with minor modifications [67]. The experiment was conducted in the greenhouse of the Institute of Botany, Jiangsu Province and Chinese Academy of Sciences (32°02′ N, 118°28′ E, elevation 30 m) under natural light. After 2 months of cultivation, the seedlings were treated with 350 mM NaCl and sampled at 0 h, 1 h, 6 h, 24 h and 48 h after salt stress with liquid nitrogen. Total RNA was extracted from leaves and roots of salt-stressed samples using the FastPure Universal Plant Total RNA Isolation Kit (Vazyme, Nanjing, China). Genomic DNA digestion and reverse transcription of the extracted RNA were performed using the HiScript III 1st Strand cDNA Synthesis Kit (+gDNA wiper) (Vazyme, Nanjing, China), followed by RT-qPCR-based expression analysis on a Jena qTower3 platform (Analytik Jena AG, Germany) using ChamQ Universal SYBR qPCR Master Mix (Vazyme, Nanjing, China). The qPCR reaction was performed in a volume of 20 µL with the following program: 95 °C for 30 s, followed by 40 cycles of 95 °C for 5 s and 60 °C for 34 s. The 2^−ΔΔ^Ct method was employed to calculate the relative gene expression levels [68]. The *ZjACT* gene was used as an internal reference gene, as in the previous study [34]. All primers used for qRT-PCR in this study are listed in the Table S3. All data are presented as the mean ± standard deviation (SD) of three independent biological replicates.

### 4.6. PPI Network Analysis

To predict the potein interactions between PUBs and other proteins of *Z. japonica*, PPI was performed with the STRING server (https://string-db.org/, accessed on 20 March 2022) [69]. The PPI network of ZjPUBs was visualized by Cytoscape v3.10.1 [70].

## 5. Conclusions

In this study, a total of 71 *ZjPUB* genes were identified, and classified into five groups based on the protein tree analysis of PUB protein sequences of rice, Arabidopsis and *Z. japonica*. ZjPUBs showed a closer relationship with OsPUBs, which is consistent with their evolutionary relationship. The length of ZjPUBs ranged from 101 to 2,967 aa. Based on the *cis*-element and PPI prediction, this study found that *ZjPUBs* might participate in the ABA signaling pathway, acting as its upstream signal and ubiquitination protein. The expression levels of some *ZjPUBs* were obviously upregulated, mainly in the roots, which indicated that *ZjPUBs* might play pivotal roles in the salt stress response of *Z. japonica*. Further studies of *PUB* genes might provide materials for the molecular breeding of zoysiagrass with high salt tolerance.

## Figures and Tables

**Table 1:** Characteristics of the *PUB* gene family in *Z. japonica*.

Gene Name	Transcript ID	CDS Length (bp)	Amino Acids (aa)	MW (Da)	pI	Subcellular Localization
ZjPUB1	Zjn_sc00010.1.g02110.1.am.mkhc	2331	777	88,914	5.35	endomembrane system
ZjPUB2	Zjn_sc00182.1.g00060.1.sm.mkhc	1398	466	52,315.6	6.02	nucleus
ZjPUB3	Zjn_sc00102.1.g00610.1.sm.mkhc	1344	448	49,809.3	5.03	nucleus
ZjPUB4	Zjn_sc00022.1.g06260.1.sm.mk	576	192	21,098.8	4.86	nucleus
ZjPUB5	Zjn_sc00041.1.g00960.1.am.mk	1200	400	44,426.1	7.1	chloroplast
ZjPUB6	Zjn_sc00049.1.g01240.1.sm.mk	1374	458	51,715.7	5.5	nucleus
ZjPUB7	Zjn_sc00049.1.g00160.1.sm.mkhc	2424	808	90,586	7.46	nucleus
ZjPUB8	Zjn_sc00067.1.g02790.1.am.mkhc	2208	736	82,274.4	7.26	nucleus
ZjPUB9	Zjn_sc00066.1.g00450.1.am.mk	2304	768	85,501.4	6.09	nucleus
ZjPUB10	Zjn_sc00102.1.g00620.1.am.mk	1764	588	65,486.1	8.06	nucleus
ZjPUB11	Zjn_sc00026.1.g00430.1.sm.mkhc	2976	992	108,074.2	6.65	chloroplast
ZjPUB12	Zjn_sc00004.1.g08300.1.am.mkhc	1464	488	53,820.9	4.55	nucleus
ZjPUB13	Zjn_sc00023.1.g01280.1.sm.mkhc	2859	953	104,355	6.38	chloroplast
ZjPUB14	Zjn_sc00150.1.g00150.1.sm.mkhc	825	275	31,037	7.17	nucleus
ZjPUB15	Zjn_sc00016.1.g06570.1.sm.mkhc	303	101	11,515.1	5.7	nucleus
ZjPUB16	Zjn_sc00093.1.g01230.1.sm.mkhc	3093	1031	112,713.5	6.61	nucleus
ZjPUB17	Zjn_sc00131.1.g00970.1.sm.mkhc	3069	1023	111,844.9	6.15	nucleus
ZjPUB18	Zjn_sc00165.1.g00170.1.sm.mkhc	1890	630	69,830.4	5.31	plasma membrane
ZjPUB19	Zjn_sc00007.1.g00090.1.cf.mkhc	903	301	34,248.6	4.84	nucleus
ZjPUB20	Zjn_sc00010.1.g03830.1.sm.mkhc	1266	422	46,902.2	5.08	nucleus
ZjPUB21	Zjn_sc00006.1.g02940.1.sm.mk	1164	388	42,146.4	5.02	nucleus
ZjPUB22	Zjn_sc00058.1.g02220.1.sm.mk	1113	371	39,634.4	8.65	nucleus
ZjPUB23	Zjn_sc00020.1.g01280.1.sm.mk	969	323	33,963.6	5.69	nucleus
ZjPUB24	Zjn_sc00003.1.g07570.1.am.mk	1578	526	56,312.6	8.25	nucleus
ZjPUB25	Zjn_sc00002.1.g09550.1.am.mk	1848	616	66,461.4	8.93	chloroplast
ZjPUB26	Zjn_sc00012.1.g05350.1.am.mk	1512	504	51,890.3	9.76	chloroplast
ZjPUB27	Zjn_sc00020.1.g01450.1.am.mk	2397	799	85,768.3	9.95	nucleus
ZjPUB28	Zjn_sc00006.1.g03530.1.am.mk	1911	637	67,282.2	9.7	nucleus
ZjPUB29	Zjn_sc00002.1.g08810.1.am.mk	1338	446	47,119.9	7.7	mitochondrion
ZjPUB30	Zjn_sc00003.1.g06760.1.am.mk	1332	444	47,189	7.88	nucleus
ZjPUB31	Zjn_sc00093.1.g00320.1.am.mk	1554	518	55,204.8	10.25	nucleus
ZjPUB32	Zjn_sc00007.1.g05420.1.am.mk	1488	496	53,359.2	8.73	chloroplast
ZjPUB33	Zjn_sc00022.1.g01940.1.sm.mk	1368	456	48,826.9	8.17	nucleus
ZjPUB34	Zjn_sc00006.1.g06155.1.br	432	144	14,928.8	6.79	nucleus
ZjPUB35	Zjn_sc00004.1.g08630.1.am.mk	1494	498	54,381.3	8.31	nucleus
ZjPUB36	Zjn_sc00023.1.g01610.1.sm.mk	1242	414	45,167.9	7.34	nucleus
ZjPUB37	Zjn_sc00007.1.g08400.1.am.mk	1449	483	52,598.4	5.33	nucleus
ZjPUB38	Zjn_sc00022.1.g04900.1.sm.mk	1287	429	46,311	4.85	nucleus
ZjPUB39	Zjn_sc00028.1.g01890.1.am.mk	933	311	33,132.5	6.7	nucleus
ZjPUB40	Zjn_sc00034.1.g05980.1.am.mk	1830	610	66,145.2	10.03	nucleus
ZjPUB41	Zjn_sc00004.1.g14050.1.sm.mk	1272	424	45,043.1	7.06	nucleus
ZjPUB42	Zjn_sc00020.1.g01520.1.am.mk	1545	515	55,689.2	9.83	nucleus
ZjPUB43	Zjn_sc00006.1.g03610.1.am.mk	1314	438	46,999.6	7.94	nucleus
ZjPUB44	Zjn_sc00071.1.g01640.1.am.mk	3036	1012	111,234.8	7.37	chloroplast thylakoid membrane
ZjPUB45	Zjn_sc04324.1.g00010.1.am.mk	678	226	23,553.7	7.64	nucleus
ZjPUB46	Zjn_sc00018.1.g06300.1.sm.mk	2091	697	73,597.5	7.31	chloroplast
ZjPUB47	Zjn_sc00047.1.g02090.1.am.mk	2241	747	81,591.7	5.39	endomembrane system
ZjPUB48	Zjn_sc00174.1.g00010.1.sm.mk	1965	655	70,775.8	6.66	nucleus
ZjPUB49	Zjn_sc00012.1.g07180.1.sm.mkhc	1923	641	68,015.4	7.07	nucleus
ZjPUB50	Zjn_sc00025.1.g05030.1.am.mk	2376	792	84,931.1	10.13	endomembrane system
ZjPUB51	Zjn_sc00009.1.g08070.1.sm.mk	2055	685	73,004.8	6.71	chloroplast
ZjPUB52	Zjn_sc00040.1.g03750.1.am.mk	2061	687	73,448.3	6.24	nucleus
ZjPUB53	Zjn_sc00090.1.g01510.1.am.mk	2430	810	87,041.7	6.28	nucleus
ZjPUB54	Zjn_sc00006.1.g06160.1.sm.mk	1368	456	49,690	5.01	nucleus
ZjPUB55	Zjn_sc00020.1.g03330.1.sm.mkhc	1377	459	49,914.2	5.94	nucleus
ZjPUB56	Zjn_sc00015.1.g04170.1.am.mk	1914	638	70,795.3	7.92	nucleus
ZjPUB57	Zjn_sc00022.1.g02290.1.sm.mkhc	1569	523	57,066.2	5.52	nucleus
ZjPUB58	Zjn_sc00184.1.g00310.1.sm.mkhc	2367	789	85,448.1	7.41	nucleus
ZjPUB59	Zjn_sc00002.1.g06310.1.sm.mkhc	2118	706	76,646.2	8.2	nucleus
ZjPUB60	Zjn_sc00038.1.g02650.1.sm.mkhc	1971	657	71,535.9	5.22	nucleus
ZjPUB61	Zjn_sc00093.1.g00050.1.sm.mk	1416	472	49,182.2	6.32	chloroplast outer membrane
ZjPUB62	Zjn_sc00018.1.g06490.1.sm.mk	1365	455	47,388.5	7.28	chloroplast
ZjPUB63	Zjn_sc00091.1.g00910.1.am.mk	2031	677	70,993.7	8.76	nucleus
ZjPUB64	Zjn_sc00039.1.g04330.1.am.mk	480	160	16,782.6	7.09	chloroplast
ZjPUB65	Zjn_sc00039.1.g04340.1.am.mk	651	217	23,010.2	5.02	chloroplast
ZjPUB66	Zjn_sc00008.1.g00320.1.sm.mk	1272	424	46,803.7	6.76	nucleus
ZjPUB67	Zjn_sc00071.1.g00460.1.am.mkhc	2496	832	90,311.8	5.76	nucleus
ZjPUB68	Zjn_sc00007.1.g07460.1.sm.mkhc	2319	773	86,679.4	5.64	nucleus
ZjPUB69	Zjn_sc00022.1.g03900.1.sm.mkhc	1884	628	69,200	6.42	nucleus
ZjPUB70	Zjn_sc00056.1.g00870.1.am.mkhc	1470	490	54,562.6	4.82	nucleus
ZjPUB71	Zjn_sc00009.1.g03620.1.am.mkhc	2373	791	86,729.6	6.14	nucleus

## Data Availability

Data are contained within the article and Supplementary Materials.

## Supplementary Materials

The following supporting information can be downloaded at: https://www.mdpi.com/article/10.3390/plants13060788/s1, Table S1: Predicted PPI network of ZjPUBs; Table S2: Protein sequence of the U-box gene family in *Z. japonica*; Table S3: Primers used in qRT-PCR.
