# Supplementary material for: Genome-Wide Identification and Expression Analysis of the PUB Gene Family in Zoysia japonica under Salt Stress
Source: Plants (Basel). 2024 Mar 10;13(6):788. doi: 10.3390/plants13060788 (PMC10974829; doi:10.3390/plants13060788)
Supplement: Supplementary file 1 [file plants-13-00788-s001.zip › Table S3.pdf]

Table S3. Primers used in qRT-PCR.

Primer name	Primer sequence（5’-3’）
ZjPUB22-F/R	CCGTCTCCTCGAGACCATC/GTAGGAGTGCCTGCCACA
ZjPUB26-F/R	CCGGCTCTCCTCTCCTTC/CCTCGGTGAGGTAAGAGACC
ZjPUB27-F/R	GTCGTTCTCGTACGTGGTTG/GTATTCTCGTTGCCCGGAAG
ZjPUB29-F/R	GGTCTGCATGATGCCGTTAG/GCGTTCATCCTTCCCTCCAG
ZjPUB30-F/R	CAGGGACGCAACTAAGGAGA/TGAGCCCTCTGAAATCCACA
ZjPUB32-F/R	GGTGGCCATCTCTGAGCTAC/GGGCAGATGAAGTGGTTTGG
ZjPUB33-F/R	TTGGTCGTGTTCTTCCCTCT/GCCATGGGCAAGGATTGAAA
ZjPUB36-F/R	TCCTTCTCAAGCAGGAGAGC/GAGATCTTGGCGAGGATGGA
ZjPUB39-F/R	GATGATGAGCGAGAGCGAAG/GTCAACCGTCGGACAAGAAT
ZjPUB42-F/R	GGCGAGCTTTCAACGAGTTT/CGAAGAGACCTCCATCGTGT
ZjPUB43-F/R	CGGCTTTGAGCCTTCTTCAT/CAGACGCTAACGTGTCCAAG
ZjPUB46-F/R	GATCGCGTTTCTCGAGGATG/TACACCAGGAAGGCCATCAG
ZjPUB47-F/R	CTCCGCAGCTTGATTTCACA/TGCAGCTACACACTGGATCA
ZjPUB48-F/R	TACAGTGGATGGAGGAGGGA/ACCACTGCGAAATCAAGCTG
ZjPUB51-F/R	GGTTCCAATCCTGACCCAGA/GGATCGCGCATAAGATCGAG
ZjPUB52-F/R	GCGTCCATCGTGGAGAAAC/CTTCGCGATGTTCTCCCTCT
ZjPUB63-F/R	GCAACACTCCATCGTTCCTG/GCTTAGCCTGGAAGGGATCA
ZjPUB64-F/R	CTTCGCCAAGTCGTCCAC/GGAAATCGGGCACACGAA
ZjActin-F/R	GCTCAACCCCAAGGCTAAC/AGAGCGTATCCCTCGTAGATG
